# Potential for use of creatine supplementation following mild traumatic brain injury

**DOI:** 10.2217/cnc-2016-0016

**Published:** 2017-03-21

**Authors:** Philip John Ainsley Dean, Gozdem Arikan, Bertram Opitz, Annette Sterr

**Affiliations:** 1 School of Psychology, Faculty of Health and Medical Sciences, University of Surrey, Guildford, Surrey, GU2 7XH, UK

**Keywords:** behavioral symptoms, brain injury, clinical outcome, concussion, creatine, magnetic resonance spectroscopy, mild TBI, neurobiology, neuroprotection, postconcussion syndrome, treatment

## Abstract

There is significant overlap between the neuropathology of mild traumatic brain injury (mTBI) and the cellular role of creatine, as well as evidence of neural creatine alterations after mTBI. Creatine supplementation has not been researched in mTBI, but shows some potential as a neuroprotective when administered prior to or after TBI. Consistent with creatine’s cellular role, supplementation reduced neuronal damage, protected against the effects of cellular energy crisis and improved cognitive and somatic symptoms. A variety of factors influencing the efficacy of creatine supplementation are highlighted, as well as avenues for future research into the potential of supplementation as an intervention for mTBI. In particular, the slow neural uptake of creatine may mean that greater effects are achieved by pre-emptive supplementation in at-risk groups.

Creatine supplementation has been predominately used for peripheral enhancement, increasing muscle function and strength in an athletic environment [1] and in those with impaired movement [2,3]. However, this molecule has also begun to be investigated for its use in cognitive enhancement in nonclinical groups [4], and as a potential neuroprotective supplement to reduce damage and alleviate symptoms in those with neurological disorders [2,3,5] and brain injury [6,7]. The interest in both muscular and neural supplementation comes from its predominant role in energy homeostasis in high or fluctuating energy demand tissues such as muscles and brain [8]. Increasing the creatine stores in the brain could therefore aid in neurodevelopmental, psychiatric and acquired injury-based disorders where there is evidence of dysfunctional energy processing or altered creatine.

This review investigates the potential for creatine supplementation following mild traumatic brain injury (mTBI), as well as potential neuroprotective effects if taken prior to injury. First, the case for a role of creatine in mTBI will be discussed, looking at overlap in biology and symptom report as well as alterations in neural creatine after mTBI. Studies investigating the effects of creatine supplementation after traumatic brain injury (TBI) will then be summarized, along with its effect in nonclinical and related clinical groups. Finally, the factors to consider for creatine supplementation, such as neural uptake, timing, dosing, individual effects and safety issues, will be presented.

## The case for a role of creatine in mTBI

### Overlap in biology

TBI induces both focal damage, where the brain impacts the skull, and more diffuse disruption in neural function. Focal injury is associated with vascular damage in cortical gray matter areas, which can lead to secondary damage around the site of impact due to ischemia, glutamate neurotoxicity, breakdown of the blood–brain barrier (BBB), edema and neuroinflammation [9,10]. The role of neuroinflammation after TBI may be beneficial as well as damaging, depending on the time period and circumstances, and is still an active area of research [11]. Large-scale focal injury is uncommon after mTBI, but there is evidence for vascular changes (reduced cerebral blood flow and capillary number) and similarities in the metabolic cascade that leads to secondary damage such as edema and neuroinflammation [12,13].

The primary mechanism for damage in mTBI is thought to be the diffuse stretching of axons by internal shear and strain forces (traumatic axonal injury [TAI]) [9,10]. These forces are unlikely to cause immediate axonal disconnection or swelling [14], and instead induce secondary damage via a metabolic cascade [15–18] of neurochemical, metabolic and inflammatory changes [19–21]. This cascade may ultimately result in delayed axonal disconnection and swelling or even cell death, but it is more probable that axons will remain intact but functionally impaired, with altered conduction velocities [14].

**Figure 1.**
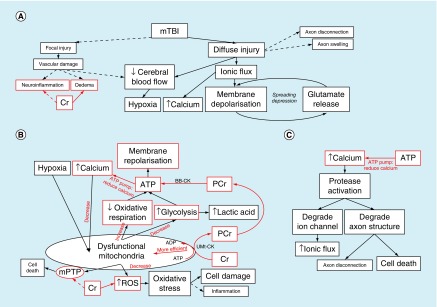
**The neurometabolic cascade after mTBI, and its overlap with creatine biology (in red).** **(A)** How diffuse injury after mTBI results in large-scale membrane depolarisation (a ‘spreading depression’-like state), reduced blood flow and increased intracellular calcium. **(B)** The generalised cellular energy crisis, where large amounts of ATP are required to repolarise the membranes and counteract the ‘spreading depression’-like state. This occurs in a low oxygen environment, with dysfunctional mitochondria (due to calcium sequestering), resulting in increased glycolysis and lactic acid formation, along with increased oxidative stress and potential formation of mPTP. **(C)** The secondary effects of increased intracellular calcium. Red boxes and arrows indicate the role and influence of creatine within mTBI neuropathology. ADP: Adenosine di-phosphate; ATP: Adenosine tri-phosphate; BB-CK: Braincreatine kinase; Cr: Creatine; mPTP: Mitochondrial permeability transition pore; mTBI: Mild traumatic brain injury; PCr: Phosphocreatine; ROS: Reactive oxygen species; uMt-CK: Ubiquitous mitochondrial creatine kinase.

In brief, the metabolic cascade [15–18] starts when the diffuse stretching of axons makes membranes more permeable and allows an ionic flux, whereby there is an increase in intracellular calcium and sodium as well as extracellular potassium. This leads to nonspecific membrane depolarization, which in turn triggers voltage or ligand-gated ion channels and an indiscriminate release of excitatory neurotransmitters such as glutamate. These neurotransmitters bind to synaptic receptors to instigate further depolarization and cause a feedback loop resulting in a ‘spreading-depression’-like state (see Figure 1A) [18]. Depolarized neurons use ATP-dependent ionic pumps to try and regain resting membrane potential, with replenishment of neuronal ATP stores requiring increased oxidative respiration and glycolysis. However, cerebral oxidative respiration is compromised by vascular changes during mTBI (reduced blood flow and impaired autoregulation) [13], and impaired mitochondrial function (due to increased intracellular calcium) [18]. Anaerobic glycolysis is increased, resulting in raised intracellular lactic acid, but may not be sufficient to provide the extra energy required. This process therefore results in a generalized cellular energy crisis (see Figure 1B) [18], with metabolic dysfunction potentially lasting for days or weeks after injury [18,20].

Part of the reason for more long-term metabolic alterations could be due to the secondary effects of impaired mitochondrial function and increased intracellular calcium. Mitochondria sequester some of the intracellular calcium, but this impairs their ability to perform oxidative respiration efficiently and results in the production of damaging free radicals and reactive oxygen species (ROS) [14,18,21]. These induce oxidative stress, causing lipid peroxidation, cytoskeleton damage and perhaps even inflammatory changes [13,14,18,22]. Furthermore, the altered metabolic environment within mitochondria (increased calcium, oxidative stress, reduced ATP) can lead to the formation of mitochondrial permeability transition pores (mPTP), which can in turn release proteins that induce cell death (apoptosis) [23]. Increased intracellular calcium not only impairs mitochondrial function, but also activates enzymes that degrade ion channels to make them more porous (increasing ionic flux), breakdown axonal structures such as the cytoskeleton and microtubules (impairing axonal transport), and potentially lead to axonal disconnection and even apoptosis (see Figure 1C) [12,18]. Finally, alterations in neurotransmission have been reported after injury [13,18,19], particularly in the cholinergic and catecholaminergic (dopamine/adrenergic) systems. There is also evidence of altered GABA neurotransmission after injury, with reduction perhaps altering the inhibitory-excitatory neurotransmission balance after injury [18].

Creatine (methylguanidoacetic acid) is a naturally occurring nitrogenous organic acid, stored predominately in skeletal muscles [24,25] but also present in the liver, kidney, testes and brain [22]. The predominant role of creatine (Cr) is to maintain energy homeostasis by keeping cellular ATP levels constant in cells with high and fluctuating energy demands, such as those found in muscles and the brain [4,8,26]. It is in this role that the overlap with mTBI neuropathology is most apparent, as the cellular energy crisis induced by mTBI places an extremely large demand on ATP in an environment of reduced blood flow and hypoxia.

Creatine kinase (CK) enzymes within the mitochondria (uMt-CK) transfer the energy stored in ATP to Cr by transferring the phosphate group to create Phosphocreatine (PCr) and ADP (see Figure 1B). In times of low energy demand, mitochondria use oxidative respiration to create ATP, with some of this energy locked away or ‘stored’ as PCr. In times of high energy demand, a CK in the cytosol (BB-CK) can regenerate ATP from these PCr stores as required. As PCr is smaller and less negatively charged than ATP, it is able to move more freely through the cell [26], diffusing quickly between the site of ATP production (mitochondria) and use (e.g., synapse) [5], where it is able to be stored in large amounts for future use [26]. This Cr/PCr/CK mechanism means that levels of ATP can remain constant even when the energy demand increases by several orders of magnitude (‘the stability paradox’ [27]), by both regenerating at site of use from local PCr stores (‘temporal energy buffer’), and quickly shuttling newly created PCr from mitochondria to the site of use (‘spatial energy buffer’) [2,5,28]. Indeed, it is so efficient in this role that ATP levels remain stable until quite severe levels of oxygen depletion [26].

Critically for the case of mTBI, the creatine system enables cells to regenerate ATP quickly, efficiently and without the need for oxygen. All of these properties would help reduce the generalized energy crisis seen after mTBI, where ATP is in high demand for restoration of membrane potentials and ion gradients (e.g., calcium), neurotransmitter release and reuptake, and intracellular transport [22,23,27]. In addition, the tight coupling of the creatine system and ATP production in mitochondria has other benefits effects on the energy status of the cell, which are relevant to mTBI neuropathology. As the phosphate from ATP is transferred to PCr, this increases ADP levels in the mitochondria, which stimulates respiration and reduces the energy required for ATP synthesis [2,4,26]. Increased use of PCr to generate ATP, and increased mitochondrial efficiency reduces the amount of glycolysis required, and thus the amount of lactic acid accumulation [3,4].

In addition to beneficial effects on cell energy status, creatine has a role in the secondary mechanisms related to mitochondrial dysfunction and increased calcium homeostasis observed after mTBI. The tight coupling of creatine and ATP in mitochondria may also reduce the likelihood of the formation of ROS, thereby having an antioxidant effect [4,7,28,29]. It is also thought that creatine may have a more direct antioxidant role as a scavenger of free radicals and reactive species [2,7]. Regeneration of ATP and reduction of free inorganic phosphate by BB-CK aids in calcium homeostasis [2] (Figure 1C), decreasing both intracellular and mitochondrial calcium. Furthermore, creatine reduces the likelihood of mPTP developing in the mitochondria by protecting against the factors involved in their formation. The Cr/PCr/CK system may also help stabilize the mPTP, preventing it from opening and releasing proteins into the cytosol to induce apoptosis [3,23,29], although this effect may be weak or indirect [7,30]. Creatine may therefore play a role in the moderation of oxidative stress, intracellular calcium and apoptotic effects in neurons after mTBI.

Finally, there is tentative evidence that creatine could potentially influence edema, inflammation and altered neurotransmission after mTBI (Figure 1A). Creatine is an osmolyte, with supplementation leading to increased water retention [23,26,29]. In this way, creatine retention or release can alter water retention and osmolarity of cells exposed to hyper- and hypo-osmotic stress (e.g., in edema or calcium influx) [3,23]. Creatine has also been ascribed a putative anti-inflammatory role, reducing the appearance of inflammation markers and perhaps dampening immune response [3,29,31]. However, the exact mechanism of this effect is unknown, and may be secondary to its effects on energy metabolism and scavenging of free radicals (see Figure 1). Lastly, there is evidence that creatine may act like a neurotransmitter, with an agonist effect on GABA receptors, and some evidence of an influence on *N*-methyl-d-aspartate receptors [4,5,23,26].

In summary, there is considerable overlap between the role of creatine and the neuropathology of mTBI (Figure 1 & Table 1). Creatine’s role in energy homeostasis, keeping ATP levels constant and increasing the efficiency of mitochondrial oxidative respiration has particular potential value during the generalized energy crisis observed after mTBI. In addition, creatine has a role in calcium homeostasis, reducing oxidative stress and potentially aiding in edema, neuroinflammation and altered neurotransmission.

**Table 1.** **Overlap between mild traumatic brain injury neuropathology, role of creatine and symptom report.**

**mTBI neuropathology^†^**	**Creatine role^‡^**	**PCS symptoms^†^**
Membrane depolarization – Ionic flux – Spreading depression	– Maintains/restores membrane potentials [27] – ‘ATP buffer’ [2]	Migraine-like symptoms (headache, dizziness, light and sound sensitivity)

Calcium influx – Impair mitochondria – Activate proteases – Possible apoptosis – Axonal transport and structure effected (cytoskeleton/microtubules)	– Plays a role in calcium homeostasis [2] – Prevents influx of calcium – ATP buffer (cell transport) [2]	PCS symptoms, specifically cognition (related to axonal dysfunction/cell death)

Impaired mitochondrial function	– Plays a role in calcium homeostasis [2] – Prevents influx of calcium – Stimulates respiration [2,26] – Prevents formation of mPTP and stabilizes mPTP, preventing pore opening [3]	Increased vulnerability (Second Impact Syndrome)

Oxidative stress – Production of ROS & free radicals	– Reduces ROS by aiding mitochondria [4] – Directly scavenges free radicals [2]	Increased vulnerability (Second Impact Syndrome)

Glutamate excitotoxicity	– Aids neurotransmission and reuptake [27] – Protects against glutamate toxicity	

Lactate accumulation	Buffers lactate accumulation by reducing glycolysis [3,4]	

Swelling/edema	Used as osmolyte in brain [26]	

Inflammation	– Putative anti-inflammatory role [3,31] – Role in reducing glutamate excitotoxicity	

Altered neurotransmission balance – Reduced GABA – Altered inhib–exit balance	Putative role as neurotransmitter for GABA and NMDA receptors [4,26]	PCS symptoms, specifically cognition (related to axonal dysfunction/cell death)

^†^Data taken from [16,18].

^‡^Data taken from [26,27].

inhib–excit: Inhibitory-excitatory; mPTP: Mitochondrial permeability transition pores; mTBI: Mild traumatic brain injury; NMDA: *N*-methyl-d-aspartate; PCS: Post-concussion syndrome; ROS: Reactive oxygen species.

### Evidence for altered creatine after mTBI

In general, the most consistent finding in the acute stage after mTBI is reduced *N*-acetyl aspartate (NAA; suggesting neuronal loss [32–34]), with some evidence of increased choline (suggesting cell turnover or loss [8]), lactate (glycolysis [35]) and myoinositol (suggesting inflammation and osmotic stress [36]). Furthermore, NAA is reduced more in those with greater postconcussion syndrome (PCS) [33], and correlates with cognitive performance [34].

However, findings for total creatine are more variable, with both increases [8,37] and decreases [38,39] observed after mTBI. This variability may be due to the areas investigated in these studies, as creatine concentration is highest in gray matter areas (cerebral cortex, hippocampus, olfactory bulb, brainstem, cerebellum, spinal cord) and lowest in white matter areas and basal ganglia [5,26]. Furthermore, there is differential expression of the creatine transporter (CRT; required for creatine uptake) along similar lines [40]. If split by tissue type, the creatine results become more understandable, with increased creatine observed in white matter areas [8], and no difference or reduced creatine in gray matter or mixed areas [38,39]. Therefore, in areas with high creatine concentration (gray matter), the general finding is either unaltered or reduced creatine [38,39]. The degree of alteration may depend on the severity of injury, or the proportion of gray and white matter in mixed areas. Furthermore, the reduced gray matter creatine (usually high) and increased white matter creatine (usually low) could indicate some breakdown in the spatial energy buffer function. Overall, these findings suggest that mTBI can directly affect neural creatine stores, and there is evidence of a correlation between creatine and cognitive performance [41], as well as time since injury [42].

There is also recent evidence for reduced creatine concentration in gray matter areas (primary motor area and dorsolateral prefrontal cortex) over the course of an American football season, even when no mTBI was recorded [43]. This reduction in total creatine was related to the number of high force (60+G) subconcussive impacts recorded [44]. These studies demonstrate a relationship between head impact and creatine concentration at a subconcussive level, and suggest a mechanism for the increased vulnerability to injury (Second Impact Syndrome, discussed in the next section [15,18,21]). In addition, the use of longitudinal data, with baseline (preseason) concentration measurements, allow firmer conclusions to be drawn about the relationship between head impact and creatine.

The relative paucity of studies into creatine differences after mTBI could be due to the fact it was thought to be stable within both clinical and nonclinical populations, and so is typically used as an additional reference within magnetic resonance spectroscopy (MRS) analyses (e.g., NAA/Cr). However, creatine concentration is not constant [26], as it depends on tissue type, amount of activity, degree of vasculature, and varies across clinical groups [26]. There is clearly a need for more research, as current findings indicate that mTBI may affect neural creatine concentration, and that the concentration postinjury is related to cognitive performance.

### Overlap in symptoms

After an mTBI, individuals commonly report a number of somatic, cognitive and emotional symptoms, collectively known as PCS. There is evidence that these symptoms may result from aspects of the neurometabolic cascade observed after mTBI. For instance, the initial ionic flux could underlie very acute PCS symptoms. It induces a ‘spreading depression’ very similar to that seen in migraine, with similar symptoms reported (headache, nausea, light sensitivity, sound sensitivity and dizziness) [18]. Some PCS symptoms may also be explained by axonal dysfunction and altered neurotransmission after mTBI. Indeed, this relationship has been observed in diffusion tensor imaging ([45–50]) and functional MRI ([51–55]) studies, with altered structure (axonal integrity) and function related to slowed cognition and reaction time. In addition, the cellular energy crisis seen after mTBI may last for a considerable time after injury, and may even be present after subconcussive injury [43,44]. This energy crisis not only impacts on somatic and cognitive function as mentioned above but also increases vulnerability to a second injury with potentially longer-lasting and more severe deficits (Second Impact Syndrome [15,18,21]). This ‘window’ of vulnerability could also mean that compensation mechanisms develop on a behavioral or physiological level, with perhaps greater effort or altered neural functioning underlying some PCS symptoms such as fatigue and headache [56].

It is clear that creatine’s role in energy homeostasis is directly associated with the cellular energy crisis, ionic flux, axonal disconnection and altered neurotransmission, which may underlie PCS symptom report after mTBI (see Table 1 for summary of biological overlap with symptom report). However, further evidence for the potential role of creatine in PCS symptom report comes from individuals with impairments in creatine metabolism. For instance, impairment in the Cr/PCr/CK system can cause predisposition to migraines [57], perhaps via a reduced ability to control for ionic flux, developing into ‘spreading depression’, similar to the state observed after mTBI. Individuals with extremely low neural creatine concentration, due to genetic alterations in creatine synthesis or transport, exhibit symptoms such as mental retardation, language delay, developmental delay, motor disorders and neurological symptoms (e.g., seizures) [4,23,58]. These are much more severe symptoms than those observed in PCS, but demonstrate the necessity of creatine for typical cognitive and motor function. Large alterations in creatine cause drastic symptoms, but this also suggests that less severe symptoms may be caused by the relatively small alterations observed after mTBI. Furthermore, and of clear relevance to this review, early use of creatine supplementation in individuals with creatine synthesis deficiencies can alleviate these symptoms, and may reduce the majority of these effects on the brain [4,23,58].

## Creatine supplementation

Creatine supplementation is predominately used to enhance muscle function in athletes [1], the military [59] and in clinical movement disorders [2,3]. However, there is also research into whether supplementation may enhance cognition in general nonclinical [4] and ageing populations [25], as well as the possibility of using it as a neuroprotective in neurodegenerative diseases (e.g., Parkinson’s disease, Huntingdon’s disease, Alzheimer’s disease, amyotrophic lateral sclerosis, Charcot-Marie-Tooth’s disease, fibromyalgia) [2,3,22,60,61], psychiatric disorders (e.g., schizophrenia, bipolar disorder, major depression, anxiety disorders and psychological stress) [5,62] and brain injury (both TBI [2–4,6] and stroke [3,4,7,22]). The extensive list of applications gives an idea of the scope of research into the neural effects of creatine supplementation. However, underneath each of these applications is the effect of creatine on cerebral energy depletion, oxidative stress and mitochondrial dysfunction [2] or evidence of altered creatine metabolism within the clinical state (e.g., psychiatric diseases [5]).

### Creatine supplementation in (m)TBI

Several reviews have suggested the applicability of creatine supplementation to TBI [3,6], and there is a strong case for the potential of creatine in mTBI (see previous section). Despite this, there are no known studies into the effect of such supplementation on mTBI, and very few studies examining its effect on TBI. However, those studies that exist show promising results, and will be discussed in this section.

In animal models, creatine supplementation prior to experimental TBI reduced cortical damage (36–50% reduction) by protecting mitochondrial function (mitochondrial membrane potential increased, lower intramitochondrial ROS and calcium, ATP levels maintained) [63] and protected against some of the effects of oxidative stress (but not seizure susceptibility) [64]. Higher levels of supplementation produced a greater degree of neuroprotection, as indicated by lower markers of secondary injury (lactate and free fatty acids) [65]. However, an *in vitro* study of rat hippocampal cells suggested that creatine supplementation may not have a direct protective effect for oxidative stress, with protective effects perhaps based on reduced glutamate and calcium excitotoxicity and increased ATP/PCr levels [66].

In clinical populations, the only studies known to date come from Sakellaris and colleagues [67–69]. These studies investigated the effects of creatine supplementation (0.4 g/kg in oral suspension for 6 months) after TBI (Glasgow Coma Scale: 3–9; within 4 h of time of injury) in children and adolescents (age: 1–18 years old) [67–69]. Administration of creatine reduced post-traumatic amnesia and improved recovery [67–69], cognitive function [67], headaches, dizziness and fatigue [68], as well as language articulation and understanding [69]. It should be noted that these studies were conducted in children, after severe to moderate TBI, in open-label trials.

Overall, these studies demonstrate that creatine supplementation after TBI causes alterations in biological markers related to mTBI neuropathology (in animal models), as well as improved outcome and reduced symptom report (in clinical studies) in areas expected from the overlap between mTBI, creatine and symptom report (see Figure 1 & Table 1). Further investigation is therefore warranted for the potential use in mTBI populations, using double-blind interventions.

### Creatine supplementation in other nonclinical & clinical groups

There are further indications of the utility of creatine supplementation for mTBI in its effects on nonclinical and clinical populations. In nonclinical populations, creatine supplementation has been shown to increase cognitive performance, in particular working memory and speed of processing, in both young and old participants [4,5,25]. Along with subjective report of attention and memory problems, objective working memory and speed of processing deficits are also commonly observed after mTBI [70]. Creatine supplementation has also been shown to reduce blood–oxygen level-dependent (BOLD) response in a functional MRI visual processing task [71]. This reduction in BOLD response may be due to increased PCr stores reducing the immediate need for increased oxygen and glucose from the blood. It also demonstrates the potential for creatine to ameliorate the altered BOLD response during cognitive tasks observed after mTBI [56]. Of particular interest to mTBI research, the effect on cognition is greatest when creatine is supplemented prior to an intervention that compromises cell energy status, such as experimental hypoxia [4,5,72]. In addition, creatine supplementation prior to experimental ischemia in animal models and *in vitro* studies resulted in reduced damage, lower lactate and higher ATP in supplemented animals [7]. This is a similar state to the cellular energy crisis after mTBI, and demonstrates strong potential for neuroprotective effects of creatine administered prior to injury. Finally, there is some evidence that creatine may improve outcome in mood disorders such as depression [5] and post-traumatic stress disorder [4], both of which are common co-morbid diagnoses after mTBI.

### Creatine supplementation summary

The literature on creatine supplementation after TBI is sparse, but those studies that do exist suggest a potentially useful role for this compound in alleviating secondary damage after injury as well as protecting against damage if administered pre-injury. The possible protective role is particularly interesting when considering that two populations with high risk of mTBI are already taking this compound to some degree – athletes [1] and the military [59]. This fact would enable longitudinal studies into ability of creatine supplementation to protect against the effects of mTBI and subconcussive injury over a season or deployment.

However, it is clear from the literature on creatine supplementation that promising findings in animal models or *in vitro* have not always been followed by conclusive findings in human participants [4], particularly in clinical populations [60]. Therefore, the use of creatine as a neural supplement still requires research, as there are many unanswered questions and many factors influencing its potential utility.

## Factors to consider for creatine supplementation

The most pressing factor influencing the efficacy of creatine supplementation is its pharmacokinetics [73]. Specifically, how much does supplementation actually increase creatine levels in the brain? Dietary creatine reaches the neurons by crossing from the gut into the bloodstream, and must then be transported across the BBB. The increase in neural creatine after supplementation would depend on the mechanisms for creatine transport (and synthesis) in the brain, the timing of administration, dosing regimen, the type and form of creatine administration, and individual factors such as age and gender. In addition, researchers must be aware of the safety considerations for supplementation.

### Creatine synthesis & transport in the brain

The majority of individuals get around half of the typical daily requirement for creatine (∼2 g/day [23,26]) from a diet that contains meat and dairy products [3,5,23,24,29], with the other half being synthesized within the body [3,5,57]. This synthesis is a two-step process occurring predominately in the kidneys and liver [5,24], but also in the brain [5,23,26]. In brief, in the first step the enzyme AGAT converts arginine and glycine into guanidinoacetatic acid, and in the second step the enzyme GAMT converts this guanidinoacetatic acid and *S*-adenosylmethionine into creatine [5,24]. Both dietary and endogenously synthesized creatine is taken up into cells using a CRT [5,24]. The final stage in the cycle of creatine is its breakdown into creatinine, which is then removed in the urine (∼1.5–2% or 2 g/day) [3,24].

CRTs at the BBB are essential for supplementary creatine to enter the brain. However, although CRT are present within neurons, oligodendrocytes and on the microcapillary endothelial cells that line the BBB, they do not seem to be present in the astrocytic feet which cover around 98% of the BBB [5,24,74]. This leaves only a small amount of surface available for creatine absorption, and may therefore mean that effects of supplementation are at most small and slow-acting. Indeed, creatine supplementation does not significantly change neural creatine concentrations, or aid symptoms, in individuals with CRT deficiencies [4,23,58], highlighting its key role in neural uptake. In contrast, individuals with intact CRT, but deficient in creatine synthesis, show marked improvements in neural creatine concentrations, as well as symptom alleviation and reduced neural damage after long-term supplementation [4,23,58]. This demonstrates that despite their relatively low expression at the BBB, CRTs are capable of absorbing creatine from the plasma in sufficient quantities over time.

Creatine synthesis is tightly regulated to keep neural levels stable, so increased uptake of dietary creatine may result in reduced endogenous synthesis as a homeostatic mechanism [24]. Evidence for the potential power of this homeostatic mechanism comes from studies looking at neural creatine concentration in vegetarians and vegans, who may receive little or no dietary creatine [24]. If dietary intake is essential, then vegetarians and vegans should have lower muscular and neural creatine. However, it seems that although muscle creatine levels are lower in vegetarians [24], neural levels are similar to omnivores [75]. It therefore appears that endogenous creatine synthesis may be able to compensate for reduced dietary intake, using homeostatic mechanisms to keep neural concentrations similar to those in omnivores.

### Neural increase of creatine after supplementation

As creatine supplementation may produce only small and slow acting effects on neural creatine concentrations, it is imperative for future studies to monitor both the neural creatine concentration (using MRS) and the clinical effects of supplementation (e.g., cognition) [5,25]. Current studies have predominately examined these factors in isolation, precluding direct investigation of the relationship between creatine concentration and clinical outcome [25]. This is particularly important because creatine supplementation may have a greater peripheral (e.g., muscular) effect in comparison to its putative role in cognitive enhancement and neuroprotection.

Indeed, creatine supplementation has been shown to increase creatine concentration in plasma [76] and muscles [61]. Despite the potentially poor BBB permeability, a few studies have also demonstrated that creatine supplementation can result in significant increases in neural creatine and PCr [72,77–79] (see Table 2). Only one of these studies looked at outcome measures, and found that creatine supplementation increases neural creatine in primary motor cortex and protected against a hypoxia-induced reduction in attention [72]. This study allows direct comparison of neural creatine alteration and behavioral performance and elegantly demonstrates the potential for creatine supplementation.

**Table 2.** **Creatine supplementation: dosing, neural creatine level, behavioral effects and study specifics.**

**Creatine dose and trial type**	**Neural creatine**	**Behavioral effects**	**Study specifics**	**Study (year)**	**Ref.**
0.4 g/kg per day in solution for 6 months (28 g/day for 70 kg person) Open-label, randomized to intervention/control	N/A	– Reduced PTA – Improved recovery, cognitive function, language – Reduced headache, dizziness, fatigue	1–18 years old Post-TBI	Sakellaris *et al.* (2006, 2008, 2012)	[67–69]

4 × 5 g/day (20 g; CrMo) for 1 week Randomized, double blind, crossover design (with placebo, washout: 5 weeks)	Total creatine increase in: Primary motor area (9.2%; 7.03 ± 1.62 vs 6.44 ± 0.90; 34–37% GM)	– Reduced deficit in attention capacity – Corticomotor excitability increased	– Nonclinical – Experimental hypoxia after creatine or placebo	Turner *et al.* (2015)	[72]

4 × 5 g/day (20 g; CrMo) for 1 week, Randomized, double blind, crossover design (with placebo, washout: 5 weeks)	Cr/NAA increase in: – Primary motor area jMRUI: (6.1%; 0.52 ± 0.06 vs 0.49 ± 0.04) – LCModel: not significant (2%; 0.51 ± 0.06 vs 0.50 ± 0.03)	N/A	Nonclinical	Turner *et al.* (2015)	[80]

A: single dose 20 g (CrMo) B: 4 × 5 g (20 g)/day for 4 weeks Longitudinal study, no control group	A: no significant change B: total creatine increase in: – Parietal GM (4.7%) – Parietaloccipital WM (11.5%) – Cerebellum (5.4%) – Thalamus (14.6%)	N/A	Nonclinical	Dechent *et al.* (1999)	[77]

Loading: 2 × 0.15/kg per day in solution (CrMo) for 1 week (2 × 10 g or 20 g/day for 70 kg person)Maintenance: 2 × 0.015/kg per day in solution for 1 week (2 × 1 g or 2 g/day for 70 kg person) Longitudinal, control group (n = 5) took sucrose	Cr/Cho increase in: Left frontal lobe (9.3%; Cr/NAA increase: 8.1%; 30.6% GM) β-ATP reduction in: 5-cm slice across orbitofrontal/occipital cortex (7.8%) No significant increase in PCr (3.4%) in same area	N/A	Nonclinical	Lyoo *et al.* (2003)	[78]

2 × 10 g/day (20 g, CrMo) in solution for 1 week Pre–post, no control group	Total creatine increase in: Hippocampus (5.3%) Reduced ATP in: Medial temporal lobe (7.4%) Striatum (8.3%) No significant change in PCr	N/A	Nonclinical	Pan & Takahashi (2007)	[79]

2 × 10 g/day (20 g) for 5 days followed by: 5 g/day for 2 days Pre-post, control group (n = 6) took maltodextrin	N/A	• Reduced BOLD response in visual areas • Better performance on memory task	Nonclinical	Hammett *et al.* (2010)	[71]

4 × 5 g/day (20 g, CrMo) for 5 days followed by: 5 g/day for 24 weeks Randomized, double blind, design with placebo (dextrose)	N/A	No change in cognition nor depression	Elderly women (60–80 years old)	Alves *et al.* (2013)	[81]

4 × 5 g/day (20 g, CrMo) as tablets for 5 days Pre-post, control group (n = 60) took glucose	N/A	Vegetarian/vegan: • Creatine improved performance on memory task (word recall) All participants (meat-eater, vegetarian/vegan): • Creatine reduced variability in reaction time task post-supplementation	Vegetarian/vegan females (n = 70) compared to omnivores (n = 51)	Benton & Donohoe (2011)	[82]

BOLD: Blood–oxygen level-dependent (functional MRI signal); Cho: Choline; Cr: Creatine; CrMo: Creatine monohydrate; GM: Gray matter; jMRUI: Magnetic resonance user interface for Java; LCModel: Linear combination model; N/A: Not applicable; NAA: *N*-acetyl aspartate; PCr: Phosphocreatine; PTA: Post-traumatic amnesia; TBI: Traumatic brain injury; WM: White matter.

It seems that identification of statistically significant changes in creatine may depend on the neural area studied. The concentration of creatine varies across neural areas [25,26], and greater magnitude increases may be observed in those areas with lower presupplementation concentrations [5,25]. Indeed, one study did demonstrate greater increase in white matter (11.5%, initially low creatine area) compared with gray matter and cerebellum (4.7 and 5.4%, initially high creatine areas) after supplementation (see Table 2) [77]. Therefore, the choice of neural area(s) to sample after creatine supplementation is an important methodological issue. The choice of analysis tool also seems to be potentially important, with one study suggesting that the jMRUI software uses a technique that is more sensitive to subtle changes in creatine compared with LCModel [80]. Measurement of creatine concentration changes after supplementation is therefore methodologically possible, but potentially challenging in terms of correct choice of acquisition parameters and analysis tools. In particular, methods development is required to enable reliable identification of more subtle concentration changes and to reduce variability in placement of MRS voxel across acquisitions.

### Timing of supplementation

As discussed previously, the results from clinical trials have been variable despite very promising findings from animal and *in vitro* models. This could be due to the timing of creatine supplementation. In animal models, creatine can be given prior to injury, resulting in an immediate neuroprotective effect. However, in clinical studies, creatine supplementation can only be started after the clinical condition has been diagnosed. This will at best be in the subacute stage (within hours) after injury, when damage will already have occurred. Furthermore, as the putative neural increase in creatine is slow, further secondary damage might occur before an effective concentration is reached. Post-injury creatine supplementation may therefore be too late to enable some of the protective effects of creatine [60], resulting in reduced efficacy in comparison to the animal models.

The timing of supplementation is therefore likely to be critical for the scale and nature of the effect it exerts. Animal models with pre-TBI supplementation show larger neuroprotective effects such as reduced cortical damage, oxidative stress (ROS) and improved mitochondrial function [63–66], whereas clinical studies with post-TBI supplementation demonstrate improved symptom report such as cognitive function, headaches and fatigue [67–69]. We would therefore predict that creatine supplementation will have much greater efficacy in reducing neural damage and moderating PCS symptoms if used as a pre-emptive neuroprotective in high-risk populations (e.g., athletes and military [1,59]), rather than a postinjury medication. These high-risk populations for mTBI are already taking creatine to some degree in order to improve muscle strength and stamina [1,59], meaning the potential neuroprotective effects of this compound could be relatively easily studied.

### Strength, length & dosing regime in creatine supplementation

Previous studies use a variety of dosing regimens for creatine supplementation, and no definitive guidelines have been set [5]. In general, traditional supplementation protocols include a short (3–7 days) ‘loading phase’ with high-dose creatine (15–20 g/day) and/or a longer duration ‘maintenance phase’ of 1–3 months (3–10 g/day) [2,3,5] (e.g., see Table 2). As neural uptake of creatine is slow, it seems that prolonged administration (several days) and/or high dose is required [2,5,7], with studies using short supplementation or doses lower than 5 g reporting no significant changes in neural creatine [4,83]. It has also been suggested that the difference in outcome between animal models and clinical trials in humans [2] may be due to the size of the dose given (several times higher in animals) [60]. However, doses of up to 40 g/day have been trailed in individuals with Huntington’s disease with little clinical effect [60], and (biological) effects are observed in animal models with doses comparable to human trials (0.3 g/kg or 21 g for 70 kg) [64].

Furthermore, there may be a U-shaped dose–response curve, where lower doses are not enough to increase neural creatine, but higher doses may actually slow the absorption of creatine [5,60]. More specifically, higher doses may saturate CRTs in the intestines causing their downregulation [5], with the majority of the dose being simply excreted [5], and may be more likely to trigger other homeostatic mechanisms such as reduction in endogenous creatine synthesis [24]. High doses of creatine may also have issues for safety in short- or long-term supplementation (see the ‘Safety of creatine supplementation’ section). For this reason, studies usually split creatine supplementation into smaller doses across the day, with some variability in the number and strength of doses (Table 2). In general, multiple small doses (e.g., 5–10 g across the day, see Table 2) are preferred, with long-term doses greater than 10 g potentially having a counter-productive effect [5].

Finally, the form of administration and type of compound also influence uptake of creatine. Orally administered creatine must be able to survive the highly acidic stomach environment (which may break it down to creatinine [3]) to be transported from the intestines (where bacteria may also break it down) into the bloodstream. The form of administration (solution, powder or capsule) influences its ability to negotiate these environments as well as the speed it is absorbed into the plasma. Creatine in solution reaches peak plasma concentration first, and all supplemental creatine peaks quicker than dietary creatine [5]. One final obstacle for creatine to negotiate after supplementation is the BBB, where the limited amount of CRT means neural uptake is small and slow-acting. A number of compounds have been trialled for their increased ability to penetrate the BBB, with cyclocreatine and PCr–Mg complex acetate both able to enter the brain even when the CRT is inactive [7]. Creatine ethyl ester also has increased cell permeability, and is stable at lower pH, increasing its stability in the stomach environment [3]. These are potentially useful compounds for future research. However, the majority of studies use creatine monohydrate in solution as part of their supplementation protocol (see Table 2).

### Interindividual variability effects on creatine supplementation

Individual variation in diet, age and gender has been suggested to influence neural creatine levels, which in turn has been suggested to influence the effects of creatine supplementation (greater effects observed in areas with lower presupplementation creatine concentration) [5,25]. As discussed previously, the differences in dietary uptake of creatine between vegetarians and omnivores exhibit no influence on neural creatine concentration [75].

Current research reports both decreased [25] and increased [5] neural creatine with increasing age. This discrepancy could be due to marginal changes over age or due to variation in neural area investigated. The effect of healthy ageing on neural creatine has not been sufficiently researched, and longitudinal data are lacking [25]. This is of particular interest as cerebral energy depletion plays a major part in brain injury and is thought to be an underlying factor in some age-related neurodegenerative diseases [2].

There is also limited evidence for gender-related differences in creatine concentration, with one study reporting lower frontal lobe PCr concentration in healthy women compared with healthy men [5]. In addition, creatine supplementation showed some benefit in female, but not male, rats in a model of depression [5]. This does suggest some interaction between gender and creatine efficacy, at least in depression, and may be due to the influence of gender-related hormones and hormone cycles [5]. As the menstrual cycle also influences PCS symptom report after mTBI [84], and given the putative neuroprotective role of progesterone [85], this is particularly relevant to the development of effective creatine applications in mTBI.

### Safety of creatine supplementation

Side effects such as muscle cramping, diarrhea, nausea, vomiting, dehydration, water retention, mood alteration and kidney dysfunction have been reported after creatine supplementation [2,5,7,60,86]. Muscle cramps and gastrointestinal distress (diarrhea, nausea, vomiting) are likely due to altered osmotic balance and dehydration [86]. As such, it is recommended to drink extra water during supplementation, make sure that the supplement is fully dissolved before ingestion, avoid single high doses (perhaps >10 g) [2,86] and avoid caffeine [5]. Creatine supplementation can cause high creatinine in the urine (due to increased creatine breakdown), which is sometimes used as an indicator of kidney dysfunction and may therefore lead to false positives in these tests [86]. However, to ensure safe supplementation, it is recommended to spread the dose out evenly throughout the day, or use slow-release creatine to reduce any possible buildup of toxic compounds.

In general, creatine supplementation in traditional doses, using pure compound, is deemed safe [7,86]. However, use of creatine with other compounds known to have an adverse effect of kidney function may be unwise [86]. Long-term supplementation at or below 5 g/day is considered safe, with more data required for higher doses, although there is no indication for safety concerns [7]. Regular recording of kidney function during high-dose creatine supplementation is recommended to help early identification of unintended side effects and provide empirical data for the safety of such a regime [86]. As discussed above (see the ‘Strength, length and dosing regime in creatine supplementation’ section), high-dose creatine may also have effects in regulation of creatine synthesis and uptake, though this should stabilize after supplementation is discontinued.

## Conclusion & future perspective

Creatine supplementation demonstrates potential as a neuroprotective when administered prior to or after mTBI. There is significant overlap between the neuropathology of mTBI, the cellular role of creatine and PCS symptom report, as well as evidence of neural creatine alterations after mTBI and subconcussive injury. Furthermore, supplementation before experimental TBI reduced markers of cellular damage and energy crisis, and supplementation after clinical TBI improved outcome and reduced symptom severity. However, despite this promise, there is no known research looking specifically at supplementation after mTBI.

Creatine is a safe, naturally occurring compound, both endogenously synthesized and present in the diet, and is widely used as a supplement to build muscle strength and stamina. The efficacy of creatine supplementation for neural effects requires greater scrutiny; its ability to increase neural creatine concentration depends on an effective dose regime as well as differences in presupplementation creatine concentration. The exact mechanism and pharmocokinetics of creatine supplementation for neural effects has yet to be fully understood. For this reason, it is essential that supplementation studies monitor neural creatine concentration as well as behavioral and clinical outcome to allow firm conclusions to be drawn.

Looking forward, there is a large gap in the literature on creatine supplementation, with future studies required to investigate its potential following mTBI. In particular, creatine supplementation may have much greater efficacy if used as a pre-emptive neuroprotective in populations at high risk of mTBI (athletes and military), rather than a postinjury medication. As these high-risk populations are already taking supplement to some degree for muscle strength and stamina, this may enable longitudinal studies investigating neuroprotection against the effects of mTBI and subconcussive injury over a season or deployment. These future studies should also bear in mind the dosing regimen and safety recommendations detailed in this review.

**Executive summary**

**The case for a role of creatine in mild traumatic brain injury**

- There is considerable overlap between the neuropathology of mild traumatic brain injury (mTBI) and the role of creatine in the brain.
- Neural creatine concentrations are altered after mTBI and subconcussive injury, with a reduction (or no change) in gray matter areas and an increase in white matter areas.
- The post-concussion syndrome symptoms reported after mTBI share some similarity to those reported in individuals with impairments in creatine metabolism, and may both be due to a shared underlying cellular energy deficit.

**Creatine supplementation**

- Few studies have looked at creatine supplementation after traumatic brain injury (TBI), with none investigating mTBI.
- However, these TBI studies demonstrate that creatine supplementation can have both neuroprotective effects in animal models (e.g., protect mitochondrial function, antioxidant effects and increased ATP) and in clinical cohorts (e.g., improve recovery, reduce headache, dizziness, fatigue, improved cognition).
- Creatine supplementation also improves cognition in nonclinical populations, outcome in anxiety and depression-related mood disorders, and has a protective effect in situations that impair cell energy status.

**Factors to consider for creatine supplementation**

- The efficacy of creatine supplementation is dependent on its ability to increase neural concentration, this increase may be small and slow due to limited creatine transporter at the blood–brain barrier.
- Other factors that affect efficacy are dosage regime (amount, doses/day, length of supplementation, type of administration, form of compound), prior neural concentration (which varies across areas and may be influenced by diet, age and gender) and tolerance (safety of administration, hydration status).
- Of particular interest, pre-emptive supplementation prior to injury (as in animal models) may have a greater neuroprotective effect than postinjury supplementation.

**Conclusion & future perspective**

- Creatine supplementation demonstrates potential as a neuroprotective both before and after TBI, but there is a gap in the literature for research into supplementation following mTBI.
- In particular, creatine may have potential as a pre-emptive neuroprotective against mTBI and subconcussive injury in high-risk populations such as athletes and military personnel.
